# Music Familiarization Elicits Functional Connectivity Between Right Frontal/Temporal and Parietal Areas in the Theta and Alpha Bands

**DOI:** 10.1007/s10548-024-01081-z

**Published:** 2024-10-04

**Authors:** Alireza Malekmohammadi, Gordon Cheng

**Affiliations:** Electrical Engineering, Institute for Cognitive Systems, Technical University of Munich, 80333 Munich, Germany

**Keywords:** Functional connectivity, Music, EEG, Familiarization, Theta band, Alpha band

## Abstract

Frequent listening to unfamiliar music excerpts forms functional connectivity in the brain as music becomes familiar and memorable. However, where these connections spectrally arise in the cerebral cortex during music familiarization has yet to be determined. This study investigates electrophysiological changes in phase-based functional connectivity recorded with electroencephalography (EEG) from twenty participants’ brains during thrice passive listening to initially unknown classical music excerpts. Functional connectivity is evaluated based on measuring phase synchronization between all pairwise combinations of EEG electrodes across all repetitions via repeated measures ANOVA and between every two repetitions of listening to unknown music with the weighted phase lag index (WPLI) method in different frequency bands. The results indicate an increased phase synchronization during gradual short-term familiarization between the right frontal and the right parietal areas in the theta and alpha bands. In addition, the increased phase synchronization is discovered between the right temporal areas and the right parietal areas at the theta band during gradual music familiarization. Overall, this study explores the short-term music familiarization effects on neural responses by revealing that repetitions form phasic coupling in the theta and alpha bands in the right hemisphere during passive listening.

## Introduction

Passive listening to music necessitates widespread functional connections between different regions of the cerebral cortex (Bhattacharya et al. 2001a; Karmonik et al. 2016). These connections are involved in general functions and processing various cognitive music-based tasks (e.g., perception, memory, attention, syntactic processing, and learning) (Koelsch 2012; Shahabi & Moghimi 2016) regardless of whether the music is familiar or unfamiliar. Since familiarity with musical sequences requires memory traces in different brain areas (Leaver et al. 2009; Malekmohammadi et al. 2023a, b), repeatedly listening to unfamiliar music in a way that listeners recognize as familiar sequences alters these functional coupling (Karmonik et al. 2016; King et al. 2018). However, it needs to be clarified when the functional coupling changes between different brain areas during gradual familiarization with initially unfamiliar music through passive listening. Previous EEG studies primarily focused on the temporal variation and power modulation of different frequency bands (Daltrozzo et al. 2010; Jagiello et al. 2019; Kumagai et al. 2018; Pereira et al. 2011; Thammasan et al. 2017). In contrast, neuroimaging studies have mainly been limited to locating brain connectivity rather than exploring other elements, such as spectral characteristics, which could provide a better understanding of how different brain areas interact. To the best of our knowledge, previous EEG and neuroimaging studies have yet to determine where and how these functional connectivity changes occur during gradual familiarization with unfamiliar music. Therefore, this study focuses on the spectral aspects of cortical brain connectivity based on short-term familiarization with unknown music. The following paragraphs present previous works on the music-based functional connectivity of neural activities recorded via neuroimaging and Electroencephalogram (EEG) techniques.

### Passive Listening to Music and Functional Connectivity: Related EEG Works

Passive listening to music influences functional connectivity in the alpha and gamma bands. Phase synchrony, a robust measure of functional connectivity, is enhanced in the gamma band during music listening (non-meaningful stimuli) compared to silence or other auditory stimuli such as listening to text (meaningful stimuli) (Bhattacharya et al. 2001b; Bhattacharya & Petsche 2001, 2005). In addition to the gamma band, non-uniform increases and decreases in synchronization were reported in the low alpha [7.5–9 Hz] and high alpha [9.5–12.5 Hz] bands while listening to four different pieces of music (Bach, Beethoven, Schonberg, and a Jazz piece) regardless of familiarity compared to the resting condition (Petsche et al. 1997). Similarly, Wu and colleagues reported that listening to three pieces of unknown Guqin music increases the synchronization in the alpha band [10–13 Hz] (Wu et al. 2013). These results, which indicate that passive music perception induces robust phase synchronization, prompt further investigations into the spectral changes in the brain's functional connectivity induced by music listening based on discrete cognitive elements.

Different musical-based cognitive elements, such as emotion influenced by unfamiliarity (Altenmüller 2002; Daly et al. 2014; Flores-Gutiérrez et al. 2007; Geethanjali et al. 2019; Shahabi & Moghimi 2016), sequence ending with familiar (regular) versus unfamiliar (irregular) chords (Akrami & Moghimi 2017; Ruiz et al. 2009), and importantly, musical expertise, change the functional connectivity of neural activity during passive listening. Being an expert (i.e., familiar with musical instruments and notes) increases connectivity between frontal–temporal and frontal-posterior areas in musicians compared to non-musicians in both theta and alpha bands (Krishna et al. 2017). Additionally, an increase in phase synchrony in the gamma band has been reported in musicians compared to non-musicians (Bhattacharya et al. 2001b; Bhattacharya & Petsche 2005) due to the effect of long-term memory on the representation of music (Bhattacharya & Petsche 2001). Increased functional connectivity between bilateral auditory-related cortices in musicians compared with non-musicians has also been shown during passive listening to vowels in the theta, alpha, and beta bands (Kühnis et al. 2014). Although previous reports have verified the superior strength of functional coupling of musicians compared to non-musicians during passive listening (González et al. 2021), their connectivity results are spectrally inconsistent. In other words, which frequency bands are specifically modulated while listening to music is not yet determined.

Even though cortical orientations of functional connectivity have been studied in previous works based on emotion, chord ending, and expertise during passive listening to music, it remains unclear how these orientations are formed based on enhancing music familiarity. In the following subsection, the possible connections identified by previous neuroimaging studies between different brain areas during listening to familiar versus unfamiliar music are explored (Castro et al. 2020; Freitas et al. 2018; Herholz et al. 2012).

### Passive Listening to Familiar Music and Functional Connectivity: Related Neuroimaging Works

Previous studies with neuroimaging techniques have not only localized neural activation corresponding to listening to familiar versus unfamiliar pieces of music (Green et al. 2018; Leaver et al. 2009; Nan et al. 2008; Peretz et al. 2009) but also explored the possible synchronicity of these localized brain activations. Although the reported brain networks related to familiarity are inconsistent due to the different paradigms, they generally emphasize the following three essential aspects:

- Listening to familiar music increases activity in the frontal-parietal network, including the dorsolateral prefrontal cortex, the supramarginal/angular gyri, and the precuneus (Castro et al. 2020; Tanaka & Kirino 2019).
- Listening to familiar music increases activity in the temporal-frontal and the temporal-parietal network, including the bilateral superior temporal, bilateral inferior and superior frontal, the right intraparietal, and left supramarginal gyri (Herholz et al. 2012; Satoh et al. 2006; Sikka et al. 2015; Zamorano et al. 2017).
- Listening to unfamiliar music activates the left insula and the right anterior cingulate cortex (Freitas et al. 2018).

### Current Study

This study investigates the effects of phase synchronization as an evaluation of functional connectivity induced by familiarization of music with three repetitions. Familiarization is one of the cognitive elements during passive listening and is considered a beneficial tool to facilitate the recovery of cognitive functions (Karmonik et al. 2016; Sarkamo et al. 2008). Moreover, exploring functional coupling during familiarization provides a better perspective on understanding the cooperation of the brain areas in memory recognition tasks.

In this paper, we explore the spectral sensor-based functional changes during gradual familiarization by listening to initially unfamiliar music sequences three times, which is sufficient to observe any possible effects (Fiebach et al. 2005; Fitzgibbon et al. 2004; Gruber et al. 2004; Gruber & Müller 2004). These changes are recorded via EEG sensors, offering significant insights into brain networks. Sensor-based functional connectivity is a practical approach to estimating relationships between cortical areas and provides unique insights into the coordination and cooperation of brain functions during music familiarization (Garcés et al. 2016; Huang et al. 2018).

Moreover, this study presents a potential feature—sensor-based connectivity—for use in similar future studies, such as real-time music learning or familiarization classification. Sensor-based connectivity offers quick computation and can be considered an appropriate criterion for this purpose. The weighted phase lag index (WPLI) is utilized in this paper to measure phase synchronization between all pairwise combinations of multichannel EEG signals. This study localizes functional connectivity in both the theta and alpha bands simultaneously between the parietal-frontal/temporal areas while passively listening to unfamiliar music through three repetitions.

## Materials and Methods

### Participants

Twenty right-handedness healthy male participants (age range 21–39 years, mean = 29.10, SD = 4.40) attended in passive listening to initially unfamiliar music. All participants had normal visual and audio conditions and were non-musicians (i.e., no background in music theory/education and had not played any musical instrument for more than seven years (Doelling & Poeppel 2015)). They signed a consent form before the experiment and were compensated 8 €/h for their participation. The Ethics Committee of the Technical University of Munich approved the study (reference number is 365/19 S).

### Stimuli

All the music excerpts were primarily selected from the classical genre, featuring purely instrumental sounds. This study utilizes various range of instruments and twenty-six different composers (e.g., Mozart, Vivaldi, Bach, Beethoven, Schubert, and Tchaikovsky). Each excerpt's beginning did not include a silent or solely gradually rising part of the instrument (e.g., piano, violin, or drum). We normalized the loudness of each excerpt to -1 dB based on matching the peaks in signals. No other manipulations were applied to keep the music excerpts as close as possible to the original ones. Additionally, participants individually adjusted the music volume by listening to the six different classical songs at the beginning of the experiment. More details about the musical dataset are mentioned in our previous work (Malekmohammadi et al. 2023a, b).

### Protocol

Participants sat on a comfortable chair, looking at the monitor while passively listening to 85 different excerpt pieces randomly for 10 s via a Sennheiser momentum-II headphone (with a 3.5 mm Jack plug). After listening, participants rated their familiarity with each piece on a scale from 1 to 3 for unfamiliar pieces (i.e., pieces they had not heard before) and from 5 to 7 for familiar pieces they might have heard a couple of times before or known well. Music was categorized as unfamiliar if participants selected 1, 2, or 3. Subsequently, we identified the 30 most unfamiliar music pieces based on participants' ratings of 1, 2, or 3. After that, these selected excerpts were shuffled and divided into ten blocks, each including three excerpts. Each block was randomly selected, and then participants listened to each excerpt of the block randomly. When participants listened to all excerpts of the block, we shuffled the excerpts (to avoid participants predicting the onset of the coming event). We repeated the playing of the excerpts two more times. Therefore, excerpts of each block were played three times in total. After listening to each musical excerpt, the participants provided feedback on their ability to mentally replay the same excerpt using a self-assessment Likert scale ranging from one (indicating poor ability to replay in their minds) to seven (indicating perfect ability to replay in their minds). In other words, they assessed how well they learned and memorized the excerpt with each repetition (Malekmohammadi et al. 2023a, b).

### EEG Recording

This paper used the same EEG data set as our previous work (Malekmohammadi et al. 2023a, b). EEG data were recorded with a Brain Products actiChamp amplifier containing 51 gel-based electrodes. All electrodes were located according to the 10–10 international system. Fpz was considered a ground electrode (placed 1.5 cm in front of the central frontal area), and the pair electrodes of TP9-TP10 were attached to behind the ears (linked mastoids) as references. Moreover, three electrodes were located in the center of each participant’s forehead and below the right and left outer canthi to record the vertical and horizontal electrooculograms (EOG) (Chamanzar et al. 2015). To prevent artifacts, participants were instructed to refrain from clenching their teeth, mumbling to themselves, or making any movements; moreover, the examiner monitored EEG data in real-time to identify useless trials and artifacts. A BrainVision actiCHamp amplifier (Brain Products GmbH, Germany) on a computer integrated with Pycorder software was employed to record the EEG signals at a sampling rate of 1000 Hz after applying a low-pass anti-aliasing filter with a cut-off frequency of 200 Hz. No high-pass filter was utilized during the acquisition. The impedance levels of all electrodes were kept below 15 KΩ throughout the experiment to have a high signal-to-noise ratio. The data were transferred via USB to a separate recording PC (Intel® Core™ i5 CPU 750@2.67 GHz).

### Data Analysis

All the data analyses are applied in the Matlab environment (R2019b) using Brainstorm (Tadel et al. 2011) (https://neuroimage.usc.edu/brainstorm/), FieldTrip (Oostenveld et al. 2011) (http://www.fieldtriptoolbox.org/), and a toolbox named “Easy Plot EEG Brain Network Matlab” to plot connectivity results.^1^ The continuous raw data were passed through a non-causal, zero-phase (forward-reverse) bandpass FIR (finite impulse response) filter with a cut-off frequency of 0.5 Hz and 80 Hz (González et al. 2021). The stopband attenuation is 60 dB, the filter order is 7252, and the low transition ranges from 0–0.5 Hz and from 80–80.5 Hz. Moreover, a zero-phase notch filter at 50 Hz was applied to remove any possible line noise. The maximum frequency of interest is 45 Hz; thus, performing a notch filter does not induce phase distortion. Additionally, the logistic infomax independent component analysis (ICA), implemented in Brainstorm, was performed to identify non-brain-related activities and remove any possible artifacts (e.g., eye-blink, eye-movement, and muscle activity) from the continuous data which the filter procedures could not eliminate. Independent components were removed if they were visually suspected as artifacts (mean = 5.80, SD = 1.93). Then, the continuous data were segmented from 0 to 10 s based on the stimulus time-locked after applying baseline (resting state) correction for each electrode and each trial with the pre-stimulus interval of −200 to −2 ms (${X}_{new\_i\in (0 to 10 s)}={X}_{i\in (0 to 10 s)}- {\overline{X} }_{(-200 to-2 ms)}$). All epochs were re-sampled from 1000 to 500 Hz. The average number of epochs across all subjects was 28.4, with a standard deviation of 6.38 for each condition.

To estimate connectivity between regions, induced responses to stimuli are calculated by event-related potential per condition and subtracted from all corresponding epochs (Rajaram 2014). The WPLI is used in this paper as a functional connectivity measurement because it is insensitive to volume-conduction artifacts of independent sources and provides a reliable picture of coordinated activities in the brain compared to classical indices (i.e., correlation or coherence) when dealing with nonlinear and nonstationary signals like EEG (Lau et al. 2012; Sanchez Bornot et al. 2018; Vinck et al. 2011). Although other methods exist that do not suffer from volume conductance effects, it is shown that the WPLI method provides the most accurate sensor-based brain functional connectivity maps compared to them (Sanchez Bornot et al. 2018; Stam et al. 2007; Vinck et al. 2011). If we define ${X}_{i}\left(f\right)$ and ${X}_{j}(f)$ as the complex Fourier transforms of the time series ${x}_{i}(t)$ and ${x}_{j}(t)$ of electrodes $i$ and $j$, respectively, then the WPLI between two electrodes $i$ and $j$ are defined as:

$$S_{{ij}} \left( f \right) = < X_{i} \left( f \right) > < X_{j}^{*} \left( f \right) >$$

$$WPLI_{{ij}}^{{raw}} \left( f \right) = \frac{{\sum\nolimits_{{k = 1}}^{{n_{{trials}} }} {Image} \left( {S_{{ij}}^{k} \left( f \right)} \right)}}{{\sum\nolimits_{{k = 1}}^{{n_{{trials}} }} {\left| {Image\left( {S_{{ij}}^{k} \left( f \right)} \right)} \right|} }}$$

where * means complex conjunction and $<>$ means expectation value. Parzen window is employed rather than Hanning (Sridevi et al. 2013), Hamming, or Tukey (Parzen 1956) to better evaluate harmonic magnitudes and phases in applying complex Fourier transforms of the time series. The WPLI was calculated among all trials (i.e., on overage of 28.4 trials per participant with a standard deviation of 6.38) for each subject, each condition, and each pair of electrodes from 4 to 45 Hz with a resolution of 0.5 Hz for the stimulated time (0 to 10 s) to avoid the effect of epoch length on estimated EEG functional connectivity (Fraschini et al. 2016). The reason for choosing all 10 s (stimulation period) is that the broad time window results in robustness to outliers and compensates for fewer trials.

### Statistical Analysis

**Fig. 1:**
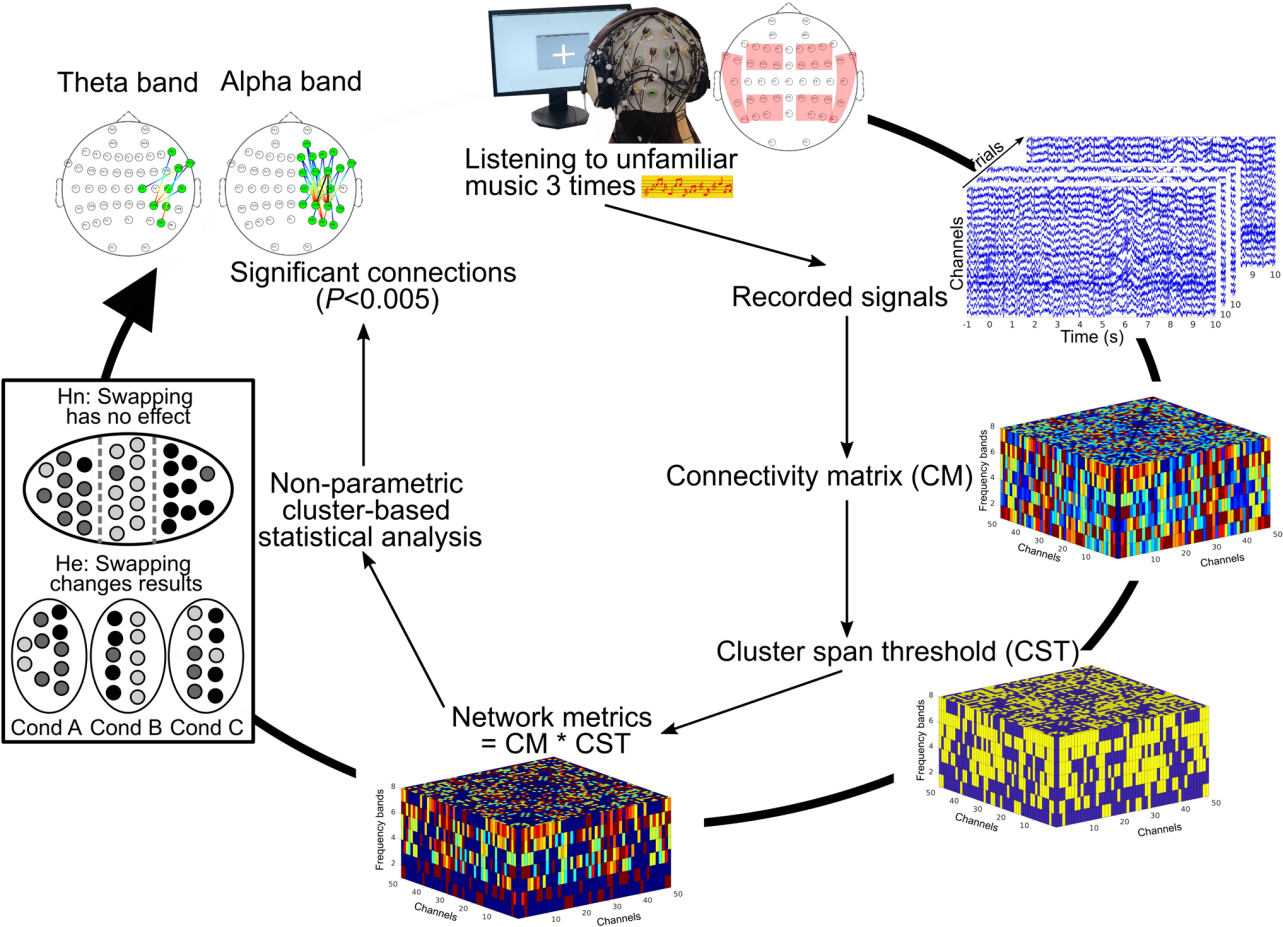
Pipeline for functional brain networks modeling and analysis: (1) Listening to unfamiliar music three times. (2) Recording EEG signals corresponding to music listening (3) Performing WPLI and creating connectivity matrix (4) Performing cluster span threshold to binarize the connectivity matrix (5) Creating network metrics by masking the connectivity matrix by cluster span threshold (6) Performing the non-parametric cluster-based statistical analysis (7) The results of connectivity analysis

The pipeline of performing statistical analysis is mentioned in Fig. 1. The procedure for performing statistical analysis is described in the following:

#### Connectivity Matrix Based on the Repeated Measure of Anova

We performed repeated measures ANOVA (*F*(2, 38)) implemented in EEGlab (Delorme & Makeig 2004) for WPLI values across three conditions (i.e., repetitions) for 2550 paired electrodes (51 electrodes * 51 electrodes – 51 self-connections = 2550) and eight frequency bands including theta [5–8 Hz], alpha [8–13 Hz], beta 1 [13–17 Hz], beta 2 [17–22 Hz], beta 3 [23–28 Hz], beta 4 [28–33 Hz], gamma 1 [33–38 Hz], and gamma 2 [38–45 Hz]. The connectivity is obtained in each frequency band by averaging the connectivity of the corresponding frequency bins (e.g., connectivity of theta band = averaging connectivity of 5 Hz, 5.5 Hz, 6 Hz, 6.5 Hz, 7 Hz, 7.5 Hz). Thus, a 3D connectivity matrix named CM (#channels × #channels × #frequency_bands) is created in a way that ${CM}_{ijk}$ shows the *F*-value of electrode $i$ and electrode $j$ in the $k$ th frequency bands. The connectivity matrix is zero for self-connections at each frequency band (i.e., connectivity of electrode $i$ and electrode $i$).

#### Network Metrics

Applying connectivity methods and extracting statistical features (e.g., *F* values) as criteria to obtain the strength of the connection between two nodes (i.e., electrodes) provides quantitive values. However, the values of CM might be influenced by numeric non-neural phenomena leading to difficulties in interpreting the results if these connections are known as significant in statistical analysis (De Vico Fallani et al. 2014). Thus, several methods are proposed such as thresholding (Van Wijk et al. 2010), Cluster Span Threshold (CST; (Smith et al. 2015)), and Minimum Spanning Trees (MST; (Stam et al. 2014)) to determine which connections belong to the network metric by creating a sparse matrix. This paper performed a sensitive and objective method named CST which employs an unbiased threshold for weighted networks to keep informative connections (Smith et al. 2015).

#### Non-Parametric Cluster-Based Statistical Analysis

In connectivity analysis, the multiple comparisons problems (MCP) is a major problem of statistical analysis due to having an extremely large number of electrode pairs which leads to not having an appropriate control for family-wise error rate (FWER) (Maris & Oostenveld 2007). Thus, this paper performed non-parametric permutation based on clustering to solve MCP to avoid suffering the results from Type I errors since the non-parametric permutation technique is based on the assumption obtained from random data generated from the original data (i.e., surrogating) rather than test statistics (Miljevic et al. 2022). In this regard, we performed 50,000^2^ permutations and applied repeated measures of ANOVA for each surrogated data. Then, we implement a one-tailed non-parametric cluster-based to find the significant connections by setting the alpha value to 0.005 (i.e., 0.01/2), which means a connection of a paired electrode has significant changes during repetitions if the corresponding *P*-value is less than 0.005.

### Post Hoc* Statistical Analysis*

#### First Post Hoc Statistical Analysis

A two-tailed paired t-test (with df = 19) was applied to WPLI values to compare functional connectivity between every two conditions: third vs. first repetition, second vs. first repetition, and third vs. second repetition. Subsequently, we performed network metrics by applying the CST method to provide a sparse matrix. To address MCP, a cluster-based non-parametric statistical test was employed, involving the creation of surrogate data through 50,000 randomizations of the original dataset across twenty subjects to correct the *P*-values. This analysis focused on 2550 electrode pairs within the theta and alpha frequency bands. A connection between two electrodes was considered significant if the corrected P-value was less than 0.005.

#### Second Post Hoc Statistical Analysis

An additional post hoc statistical analysis was performed on the functional connectivity results across each frequency band, focusing on the regions of interest. As discussed in the introduction, previous studies indicated the possible cooperation of the frontal-parietal and frontal–temporal network (Castro et al. 2020; Tanaka & Kirino 2019). Thus, we defined two bilateral frontal areas, two bilateral parietal areas, and two bilateral temporal areas as regions of interest (ROIs): “Right frontal” corresponding with electrodes F2, F4, F6, FC2, FC4, FC6; “Left frontal” corresponding with electrodes F1, F3, F5, FC1, FC3, FC5; “Right parietal” corresponding with electrodes CP2, CP4, P4, P6, CP6; “Left parietal” corresponding with electrodes CP1, CP3, P3, P5, CP5; “Right temporal” corresponding with electrodes FT10, FT8, T8, TP8, P8; “Left temporal” corresponding with electrodes FT9, FT7, T7, TP7, P7. The connectivity results of each paired electrode from each paired area are pooled. For example, to obtain the pooled connectivity of the right frontal and right parietal areas, the connectivity results of all combinations of “Right frontal” electrodes (Fz, F2, F4, F6, AF4) and “Right parietal” electrodes (CP2, CP4, P4, P6, CP6) are pooled. Then, repeated measures ANOVA (*F*(2, 38)) is applied to statistically differentiate pooled connectivity results of each two paired areas across all three conditions. Since the total number of tests is 96 (eight frequency bands * (four parietal-frontal connections + four temporal-frontal connections + four temporal-parietal connections)), performing the FDR method is sufficient to correct the obtained *P*-values for MCP. A connection between two areas in a specific frequency band is considered significant after performing FDR if the *P*-value < 0.05.

### General Linear Model: A Statistical Relationship Between Participants’ Behavioral and Brain Connectivity

To validate any potential links between the participants' behavioral feedback and changes in functional connectivity during music familiarization, a General Linear Model (GLM) was employed. Participants' feedback reflected their ability to mentally play or recall music excerpts, providing insight into their learning and memorization of the excerpts. Therefore, the GLM was applied to examine connectivity between each of the 12 paired ROI and each significant frequency band (i.e., theta and alpha band), aiming to identify direct relationships between changes in behavioral and functional connectivity across repetitions. The GLM was implemented in Matlab using the default parameters (Malekmohammadi et al. 2023a, b), assuming a normal distribution with a linear link function (identity function: $f\left(\mu \right)= \mu$), and excluding the constant term to yield a *P*-value (*P* < 0.05). Given that a total of 24 statistical GLM analyses were performed (two frequency bands * (four parietal-frontal connections + four temporal-frontal connections + four temporal-parietal connections)), FDR was performed to correct the *P*-values for MCP. A significant linear relationship between changes in behavioral and functional connectivity for the specific paired ROI and frequency band was determined if the *P*-value was less than 0.05.

## Results

### Results of Repeated Measures ANOVA

**Fig. 2:**
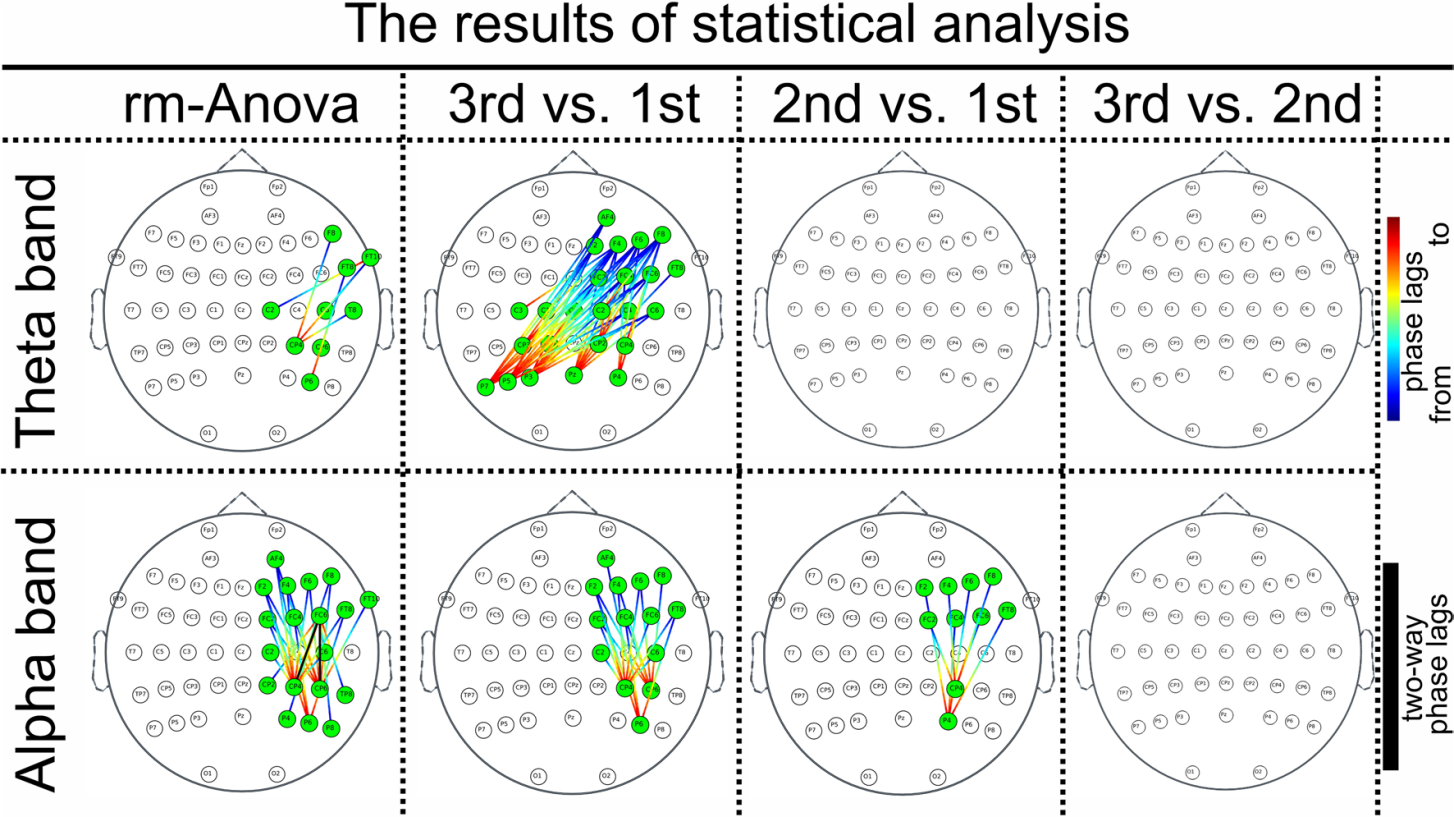
Connectivity results of repeated measures of ANOVA for significant frequency bands and the results of post hoc analysis for theta and low-alpha bands between every two repetitions: The existence of significant connections during familiarization with music mainly between the right temporal and the right parietal electrodes in the theta band and mainly between the frontal and parietal electrodes in the alpha band. Significant connectivity occurs for the third repetition compared to the first repetition between the frontal/temporal and parietal electrodes in the theta band. Moreover, significant connectivity occurs in the third repetition compared to the first and second repetitions between frontal and parietal electrodes in the alpha band

Figure 2 indicates the existence of significant connections in the theta and alpha bands as measured by the WPLI method, determined by performing repeated measures of ANOVA to study the effect of repetitions and familiarization with passive listening to unknown musical excerpts. Table 1 shows the corresponding statistical results of the pair electrodes, including *F*-values (*P*-values < 0.005) for all significant frequency bands. As shown in Fig. 2, significant connections appear between the right temporal electrodes (i.e., FT10 and T8) and the right parietal electrodes (i.e., CP4, CP6, and P6) as well as between the right frontal electrodes (i.e., F8) and the right parietal electrodes. In addition, Fig. 2 shows significant connections are observed in the alpha band between the frontal electrodes (i.e., F2, F4, F6, F8, FC2, FC4, and FC6) and the parietal electrodes (CP4, CP6, and P6). Nothing was found significant in other frequency bands for the WPLI method (*P*-values > 0.005). The following section considers the results of post hoc analysis for significant frequency bands i.e., theta and alpha bands.

**Table 1:** Statistical results of repeated measures ANOVA for WPLI methods

Repeated measures ANOVA							
Electrodes	F-values	Electrodes	F-values	Electrodes	F-values	Electrodes	F-values
Theta band: method is WPLI (*P* < 0.005)							
FT8 — CP6	4.384	C2 — FT10	6.060	T8 — CP4	5.970	F8 — CP4	4.351
C6 — FT10	4.557	FT8 — P6	5.373	FT10 — CP4	4.465		
Alpha band: method is WPLI (*P* < 0.005)							
FT10 — CP6	3.896	AF4 — CP6	4.992	FT8 — P6	9.229	C2 — CP4	4.827
FC6 — CP6	6.772	P4 — FC6	5.882	F6 — P6	5.600	FC4 — CP4	9.941
FC2 — CP6	4.411	P8 — FC6	4.207	F2 — P6	5.955	FT8 — CP4	5.043
F4 — CP6	5.634	CP2 — FC6	4.013	AF4 — P6	5.817	F6 — CP4	5.508
F8 — CP6	5.207	CP4 — FC6	7.789	FC2 — CP4	7.397	F2 — CP4	9.269
FC4 — CP6	6.383	TP8 — FC6	4.033	F4 — CP4	7.031	AF4 — CP4	5.236
FT8 — CP6	6.574	C6 — FC6	7.402	F8 — CP4	4.301	CP6 — FC6	6.772
F6 — CP6	5.527	FC4 — P6	6.103	C6 — CP4	7.143	FC6 — CP4	7.789
F2 — CP6	6.300						

### Results of Post Hoc Analysis for *Theta* and Alpha Bands

Figure 2 also presents the results of post hoc analysis between each pair of repetitions for the theta and alpha bands, highlighting significant connections identified using the WPLI method. Table 2 provides the corresponding statistical results of each electrode pair i.e., t-values (*P*-values < 0.005) for all the frequency bands between every two repetitions. As shown in Fig. 2, significant connections in the theta band occur in the third repetition compared to the first repetition between the bilateral parietal electrodes (i.e., CP2, CP4, P4, Pz, CP1, CP3, P3, P5, and P7) and both the right frontal electrodes (i.e., F2, F4, F6, F8, FC2, FC4, and FC6) and the right temporal electrodes (i.e., FT10 and FT8). Additionally, significant connections occur in the alpha band in the third and second repetitions compared to the first repetition between the right frontal electrodes (i.e., F2, F4, F6, F8, FC2, FC4, and FC6) and the right parietal electrodes (i.e., CPz, CP4, CP6, P4, P6, and P8). No significant connections were found in the theta and alpha bands when comparing the third repetition to the second repetition.

**Table 2:** Statistical results of post hoc analysis of WPLI for theta and alpha bands

Post hoc analysis for theta and low-alpha bands							
Electrodes	t-values	Electrodes	t-values	Electrodes	t-values	Electrodes	t-values
Theta band: method is WPLI (3rd vs. 1st) (*P* < 0.005)							
F4 — C3	2.888	FC6 — P3	3.390	F4 — P7	4.009	FC2 — CP3	2.854
FC2 — CP1	2.816	FC2 — P3	3.550	F8 — P7	2.996	F4 — CP3	3.217
F4 — CP1	3.131	F4 — P3	3.936	F6 — P7	3.640	FC6 — P5	2.929
FC6 — Pz	3.524	F8 — P3	3.389	F2 — P7	3.908	F4 — P5	3.555
FC2 — Pz	3.124	C6 — P3	3.466	AF4 — P7	3.229	F8 — P5	3.345
F4 — Pz	3.363	C2 — P3	3.772	F8 — P4	3.039	C6 — P5	3.239
F8 — Pz	2.808	FC4 — P3	4.532	FC4 — P4	3.148	FC4 — P5	3.120
C6 — Pz	2.845	F6 — P3	3.413	F6 — P4	2.907	F6 — P5	3.660
C2 — Pz	3.290	F2 — P3	3.232	F4 — CP2	2.740	F2 — P5	2.996
FC4 — Pz	4.171	AF4 — P3	2.921	F8 — CP2	3.321	AF4 — P5	3.330
FT8 — Pz	2.772	C4 — P7	2.883	FC4 — CP2	3.732	F8 — CP4	3.687
F6 — Pz	2.837	FC6 — P7	2.986	F6 — CP2	2.791	FT10 — C2	3.237
Cz — P3	2.905	FC2 — P7	3.280	F4 — C1	2.882	F8 — C2	2.952
C4 — P3	3.492						
Alpha band: Method is WPLI (3rd vs. 1st) (*P* < 0.005)							
FC6 — CP6	3.666	F6 — CP6	3.028	AF4 — P6	3.158	C2 — CP4	2.928
FC2 — CP6	2.830	F2 — CP6	3.076	FC6 — CP4	4.002	FC4 — CP4	5.059
F4 — CP6	3.118	FC4 — P6	3.476	FC2 — CP4	3.779	FT8 — CP4	3.411
F8 — CP6	2.969	FT8 — P6	4.141	F4 — CP4	3.813	F6 — CP4	3.001
FC4 — CP6	3.922	F6 — P6	3.150	C6 — CP4	2.961	F2 — CP4	3.987
FT8 — CP6	3.430	F2 — P6	3.093				
Alpha band: Method is WPLI (2rd vs. 1st) (*P* < 0.005)							
FC4 — P4	3.003	F6 — P4	2.739	FC6 — CP4	3.276	F4 — CP4	3.054
FT8 — P4	3.074	F2 — P4	2.968	FC2 — CP4	2.986	F8 — CP4	3.059

### *Results of *Post Hoc* Connectivity Analysis Between Different ROIs*

**Fig. 3:**
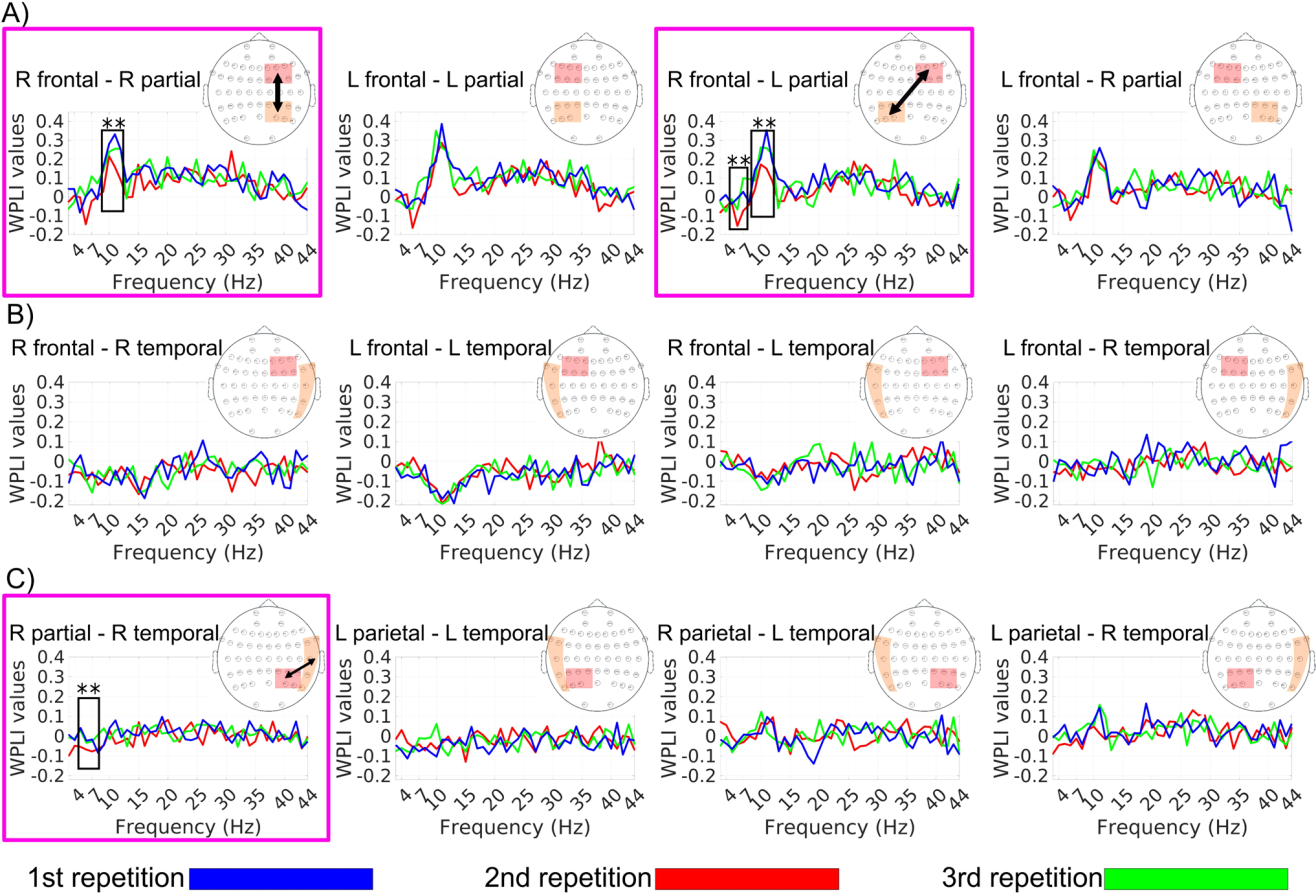
The post hoc results of connectivity analysis between the paired ROIs for the frequency bands: Increased connectivity occurs at the alpha band between the left frontal and left parietal areas in the third repetition compared to the first and second repetitions (*P* < 0.05). Moreover, increased functional connectivity occurs at theta and alpha band between the right frontal and left parietal areas in the third and second repetitions compared to the first repetition (*P* < 0.05). Finally, increased connectivity occurs at the theta band between the right temporal and right parietal areas in the theta band (*P* < 0.05)

Figure 3a shows the functional connectivity between the bilateral frontal and bilateral parietal areas. The results from this figure reveal two key findings: First, functional connectivity is significantly stronger in the third and second repetitions compared to the first repetition between the right frontal and right parietal areas in the alpha band (*F*(2, 38) = 7.2029, *P* = 0.0022). Second, functional connectivity is also significantly stronger in the third and second repetitions compared to the first repetition between the right frontal and left parietal areas in both theta band (*F*(2, 38) = 4.3378, *P* = 0.0201) and alpha band (*F*(2, 38) = 4.2003, *P* = 0.0225). Figure 3b shows that no significant connections were found between the bilateral frontal and bilateral temporal areas during short-term familiarization with music. Additionally, Fig. 3c depicts functional connectivity between the bilateral parietal and bilateral temporal areas, indicating increased functional connectivity in the third and second repetitions compared to the first repetition between the right parietal and right temporal areas in the theta band (*F*(2, 38) = 4.5626, *P* = 0.0168).

### Results of the General Linear Model (GLM)

Table 3 shows the statistical results obtained using GLM to assess any relationship between increased functional connectivity during repetitions and participants' increased familiarization with music (as indicated by subjective feedback) in every two conditions (i.e., 3rd repetition vs. 1st repetition, 3rd repetition vs. 2nd repetition, and 2nd repetition vs. 1st repetition). The results reveal a positive linear statistical relationship in the theta band between increased familiarity with unknown music and increased functional connectivity between the right frontal and left posterior areas, with significant findings in both the 3rd repetition compared to the 1st repetition (*P* = 0.0074) and the 2nd repetition compared to the 1st repetition (*P* = 0.0170). Additionally, Table 3 shows a positive linear statistical relationship in the alpha band between increased familiarity and increased functional connectivity between the right frontal and both the right posterior (*P* = 0.0038) and left posterior (0.0188) areas.

**Table 3:** Statistical analysis of GLM between functional connectivity and behavioral data

Theta band (*P* < 0.05)						
	Right frontal – right posterior	Left frontal – left posterior	Right frontal – left posterior	Left frontal – right posterior	Right frontal – right temporal	Left frontal – left temporal
3rd rep – 1st rep	*P* = 0.0436	*P* = 0.0803	*P* = 0.0074	*P* = 0.3985	*P* = 0.9725	*P* = 0.3563
2nd rep – 1st rep	*P* = 0.0272	*P* = 0.0805	*P* = 0.0170	*P* = 0.5341	*P* = 0.7694	*P* = 0.2277
3rd rep – 2nd rep	*P* = 0.6117	*P* = 0.8861	*P* = 0.8230	*P* = 0.9700	*P* = 0.6619	*P* = 0.9114

	right frontal – left temporal	left frontal – right temporal	right posterior – right temporal	left posterior – left temporal	right posterior – left temporal	left posterior – right temporal
3rd rep – 1st rep	*P* = 0.3857	*P* = 0.7821	*P* = 0.4290	*P* = 0.7022	*P* = 0.8841	*P* = 0.2453
2nd rep – 1st rep	*P* = 0.1225	*P* = 0.2922	*P* = 0.0224	*P* = 0.8535	*P* = 0.5479	*P* = 0.0982
3rd rep – 2nd rep	*P* = 0.5750	*P* = 0.7595	*P* = 0.0983	*P* = 0.6497	*P* = 0.7559	*P* = 0.9297

Alpha band (*P* < 0.05)						
	right frontal – right posterior	left frontal – left posterior	right frontal – left posterior	left frontal – right posterior	right frontal – right temporal	left frontal – left temporal
3rd rep – 1st rep	*P* = 0.0038	*P* = 0.1702	*P* = 0.0188	*P* = 0.6114	*P* = 0.2307	*P* = 0.7651
2nd rep – 1st rep	*P* = 0.0571	*P* = 0.4328	*P* = 0.0804	*P* = 0.6563	*P* = 0.2210	*P* = 0.8025
3rd rep – 2nd rep	*P* = 0.1690	*P* = 0.7189	*P* = 0.5800	*P* = 0.9291	*P* = 0.7918	*P* = 0.9681

	right frontal – right posterior	left frontal – left posterior	right frontal – left posterior	left frontal – right posterior	right frontal – right temporal	left frontal – left temporal
3rd rep – 1st rep	*P* = 0.9706	*P* = 0.5740	*P* = 0.1090	*P* = 0.8826	*P* = 0.3003	*P* = 0.3219
2nd rep – 1st rep	*P* = 0.4672	*P* = 0.7035	*P* = 0.2946	*P* = 0.6128	*P* = 0.3543	*P* = 0.2649
3rd rep – 2nd rep	*P* = 0.7151	*P* = 0.6103	*P* = 0.1839	*P* = 0.7731	*P* = 0.5941	*P* = 0.5989

## Discussion

### Functional Connectivity in the Right Hemisphere

Our results show localized coordination of phase synchronization during short-term familiarization with music, primarily in the right hemisphere areas of the non-musician participants’ brains in both the theta and alpha bands. There are three reasons why phase synchronization is localized in the right hemisphere.

Firstly, neural changes occurred in the right hemisphere because the participants were non-musicians. Neuroimaging findings demonstrate that non-musicians exhibit higher node degrees in the right hemisphere compared to musicians (Alluri et al. 2017). In other words, the right hemisphere shows greater activity than the left when a non-musician listens to music (Hirshkowitz et al. 1978). Non-musicians also exhibited increased functional connectivity in the right hemisphere during the learning process (Elmer et al. 2018), supporting the idea that our findings offer a physiological representation of a well-defined instance of human learning through short-term familiarization.

Secondly, the activation in the right hemisphere could be attributed to the nature of our nonlinguistic auditory stimuli, which is music. According to the classical perspective in auditory neuroscience, the left hemisphere is responsible for speech processing, while the right hemisphere is responsible for music processing (Ara & Marco-Pallarés, 2021; Klostermann et al. 2009). The activity lateralization to the right hemisphere during short-term familiarization with music aligns with previous studies, indicating right hemisphere dominance in music perception (Daly et al. 2014; Herholz et al. 2012).

Thirdly, prior research has suggested that the left hemisphere is specialized in processing temporal information, while the right hemisphere is specialized in processing spectral information (Zatorre et al. 2002). However, contradictory findings indicate that neural fluctuations over shorter timescales (e.g., gamma range) are associated with left hemisphere activity, while fluctuations over longer timescales (e.g., theta range) are linked to the right hemisphere (Giraud et al. 2007; Hickok & Poeppel 2007). Our findings support both theories. We revealed spectral characteristics in the right hemisphere consistent with the first theory. Additionally, we observed neural changes in the theta and alpha bands in the right hemisphere, which align with the second theory.

### Functional Connectivity in the *Theta* Band

As shown in Fig. 2, Fig. 3a, and Table 3, increased functional connectivity was found between the right frontal and the left parietal electrodes, as well as between the right temporal electrodes (FT10, FT8, and T8) and the right parietal electrodes (CP4, CP6, and P6) in the theta band during the third repetition compared to the first repetition. Since participants were initially unfamiliar with the musical excerpts, phase synchronization was weak while listening to the first repetition. However, synchronization in the theta band becomes stronger during the second and third repetitions (i.e., more familiar with music excerpts) compared to the first repetition (i.e., unfamiliar with music excerpts) (see Fig. 3a). It is shown that the increased theta synchronization related to cognitive functions connected to the mechanisms of encoding information and forming memories (Begus & Bonawitz 2020; Elmer et al. 2018). In other words, theta rhythms are linked to the learning process. This supports the physiological correlation in our study between increased theta synchronization and higher participant scores related to learning music excerpts during familiarization.

The significant enhancement in functional connectivity between frontal/prefrontal and parietal electrodes during gradual familiarization suggests temporary retention of information in working memory (Fuster 1995). Repetitions facilitate memorization (Xue et al. 2010) and result in easier retrieval of information (Atikah & Rezki 2018); thus, the phase synchronization during gradual familiarization plays a role in the interaction between the frontal/prefrontal cortex (where music sequences are continuously updated) and the parietal cortex (where music sequences are supposed to be stored) (Sarnthein et al. 1998). In other words, short-term familiarization with initially unknown music points toward functional connectivity between working and long-term memory through phase-related synchronization in the theta band. This assumption is in line with previous studies on neural responses during listening to familiar or unfamiliar music, indicating that the right frontal areas (corresponding to electrodes F2, F4, F6, FC2, FC4, and FC6) are mainly associated with working memory (Akiyama et al. 2017; Schneiders et al. 2012), while the parietal areas (CP4, CP1, CP3, CP5, P3, and P5) are linked to the long-term memory (Klostermann et al. 2009; Leaver et al. 2009; Plailly et al. 2007).

Additionally, temporoparietal theta synchronization is activated while listening to familiar music rather than unfamiliar music. Neuroimaging studies explored how imagining or listening to music activates not only the auditory cortex but also other brain areas, including the parietal cortex (Halpern 1999; Herholz et al. 2012; Zatorre et al. 2010). The phase synchronization between the temporal and parietal electrodes during music listening reflects the coordination and integration of basic auditory processing in the auditory cortex with higher-level cognitive functions in the parietal cortex, such as memory retrieval. This is because the parietal region is responsible for manipulating stored music information (Zatorre et al. 2010).

### Functional Connectivity in the Alpha Band

Previous studies have shown that exposure to music, as opposed to noise or silence, increases phase coherence in the alpha band between the parietal, temporal, and occipital regions, as well as between the prefrontal and frontal regions (Wu et al. 2012). Researchers concluded that changes in the alpha band are linked to heightened attention, which is necessary during music listening compared to noise listening or silence (Wu et al. 2012). Increased phase coherence during music listening, particularly in the high alpha range (10–13 Hz), can be observed even when the music excerpts are completely unfamiliar to listeners (Wu et al. 2013). Extending previous results, our findings in Fig. 1 and Fig. 2a illustrate an increase in functional connectivity in the alpha band during short-term familiarization with unfamiliar music between the right frontal and right parietal areas. In other words, our results show that not only does listening to unfamiliar music lead to superior phase coherency in the alpha band, but this phase coherency also increases as listeners become more familiar with the music over a short-term period.

Oscillatory changes in the lower alpha band [8–10 Hz] are related to attention, and oscillatory changes in the higher lower [10–12 Hz] alpha band are related to sensory-semantic information (Klimesch 1999). Synchronization in the alpha band plays a fundamental role in auditory information processing, such as music (Lehtela et al. 1997; Weisz et al. 2011). Therefore, the increased functional connectivity in the second and third repetitions compared to the first repetition from frontal electrodes to parietal electrodes (see Fig. 3) reflects the positive effect of familiarization on general musical information processing during passive listening to music excerpts (Flores-Gutiérrez et al. 2007).

Our results show that increased phase synchronization in the alpha band between the right frontal areas and the bilateral parietal areas (especially the right hemisphere) statistically has a linear relationship with familiarization with music (see Table 3). Given that enhanced coherent oscillations in the parietal areas might be related to the sensory stage of auditory information processing (Flores-Gutiérrez et al. 2009), we suggest that the phase synchronization related to familiarization with music at the alpha band between the frontal and parietal areas influences attention and sensory-semantic information to music.

## Conclusion

In sum, our results show that familiarization with unknown music elicits three main functional connections mainly in the right hemisphere: (1) increased phase synchronization between the right frontal and the parietal areas in the theta band, (2) increased phase synchronization between the right frontal and the right parietal areas in the alpha band, and (3) increased phase synchronization between the right temporal to the right parietal in the theta band.

## Data Availability

The data supporting the findings of this study will be openly available in [https://github.com/MalekAlireza/familiarizationWithMusic] at DOI: 10.5281/zenodo.4358978

## Footnotes

1 Johann (2021). Easy Plot EEG Brain Network Matlab (https://www.mathworks.com/matlabcentral/fileexchange/57372 easy-ploteeg-brain-network-matlab), MATLAB Central File Exchange. Retrieved February 7, 2021.

2 We also obtained the same results even with 5000 permutations.

## Declarations

### Research Involving Humand and Animal Participants

A consent form was signed by all individual participants before the experiment. All volunteers received reasonable monetary compensation for their participation.
